# Stillbirth Among Patients With Diabetes in Pregnancy in Ethiopia: Systematic Review and Meta-Analysis

**DOI:** 10.3389/fped.2021.634670

**Published:** 2021-08-05

**Authors:** Demeke Mesfin Belay, Wubet Alebachew Bayih, Abebaw Yeshambel Alemu, Amare Simegn Ayele, Demewoz Kefale Mekonen, Binyam Minuye Birhane

**Affiliations:** ^1^Department of Pediatrics and Child Health Nursing, College of Health Sciences, Debre Tabor University, Debre Tabor, Ethiopia; ^2^Department of Maternity and Neonatal Health Nursing, College of Health Sciences, Debre Tabor University, Debre Tabor, Ethiopia; ^3^Department of Midwifery, Reproductive Health, Debre Tabor University, Debre Tabor, Ethiopia

**Keywords:** diabetes mellitus, Ethiopia, mothers, stillbirth, systematic review

## Abstract

**Purpose:** Maternal diabetes mellitus and the resulting adverse fetal outcomes including stillbirth in low- and middle-income countries (LMICs) are high. Thus, setting specific evidence is pivotal to plan, evaluate, and improve national preventive measures and to achieve international sustainable development goals. Therefore, this systematic review and meta-analysis was the first of its kind to estimate the pooled prevalence of stillbirth and its determinants among diabetic mothers in Ethiopia.

**Methods:** Primary studies were exhaustively searched using PubMed, ScienceDirect, Web of Science, SCOPUS, and Google Scholar databases, and gray literature found in Addis Ababa and Haramaya University online repositories was accessed. Eligible studies were selected and critically appraised for quality using the Joanna Briggs Institute (JBI) quality appraisal checklist. The overall prevalence of stillbirth among diabetic mothers was estimated using a weighted inverse random-effect model. *I*^2^ statistic was used for evidence of heterogeneity. Egger's test and funnel plot were used to check the presence of publication bias.

**Results:** The pooled prevalence of stillbirth among diabetic mothers was 2.39 [95% confidence interval (CI): −0.20, 4.97]. Being a housewife [adjusted odds ratio (AOR) = 2.25; 95% CI: 1.26, 3.23], maternal age of <30 years [AOR = 2.08 (95% CI: 1.02, 3.13)], and gestational age of <37 completed weeks [AOR = 9.76 (95% CI: 7.83, 11.70)] increased the risk of stillbirth among diabetic mothers.

**Conclusions:** The national pooled prevalence of stillbirth among diabetic mothers was 2.39%. Maternal age of <30 years, gestational age of <37 completed weeks, and being a housewife were significantly associated with stillbirth.

**Trial registration:** PROSPERO 2020: CRD4202016774.

## Introduction

Diabetes mellitus is a metabolic syndrome characterized by hyperglycemia due to either deficiency in insulin secretion or reduced insulin action (1). Currently, diabetes mellitus is a severe public health problem worldwide. Maternal diabetes mellitus in low- and middle-income countries (LMICs) is high, which accounts for 90% (2). Thus, maternal hyperglycemia complicates 17% of pregnancy, which results in different adverse birth outcomes for both the mothers and newborns (3–5). Maternal hyperglycemia has many adverse effects on embryogenesis and fetal development (6) like stillbirth (7). According to the World Health Organization (WHO), stillbirth can be defined as a baby born with no sign of life at or after 28 weeks of gestation (8).

Pre-existing maternal diabetes during pregnancy had four to five times higher risk of stillbirth (9). On the other hand, the rate of stillbirth among type 1 and type 2 diabetic pregnant women were 16.1 and 22.9 per 1,000 births, respectively (10). Similarly, a systematic review conducted in LMICs indicated that the incidence of stillbirth was 6.3% higher among diabetic pregnant mothers than non-diabetic pregnant mothers (11). Elsewhere, the rate of stillbirth among diabetic pregnant mothers in different parts of the world is as follows: 13.9 per 1,000 births in England (12), 25% in Australia (13), 3.66% in Saudi Arabia (14), 2.6% in King Khalid University Hospital (4), 2% in Nigeria (15), 5.2% in Ghana (16), and 2.3% in Cape Town (17). Moreover, studies conducted in different parts of Ethiopia showed that the magnitude of stillbirth among diabetic pregnant mothers ranges between 2.6 and 16.05% (18–23).

Stillbirth can result in long-lasting disability, which may have significant burdens on parents, families, healthcare providers, and societies worldwide (24). Elsewhere, the loss of life and the psychological and financial costs for women, families, and societies are severe and long-lasting (25). According to a 2015 WHO report, more than 7,178 deaths per day resulted from stillbirth. The majority (98%) of this death occurred in LMICs (26).

Factors such as higher HbA1c and maternal overweight/obesity increase the risk of stillbirth among diabetic pregnant mothers (10). In addition, lack of antenatal, medical, and preconception cares are factors affecting stillbirth among diabetic pregnant mothers (2, 27, 28).

Prevention of stillbirth has been recognized as an essential part of the Sustainable Development Plan (SDP), intended to end preventable stillbirth-related death by 2030 (29). The Every Newborn Action Plan aims to end preventable deaths, setting a stillbirth target of 12 per 1,000 births or less by 2030 (8). High-income and upper-middle-income countries have already met this target, but 56 countries particularly in Africa will have to more than double the present progress to reach this target (24). In addition, the International Federation of Obstetrics and Gynecology (FIGO) and the International Federation of Diabetes (IDF) issued a joint statement and declaration outlining the public health challenge posed by hyperglycemia in pregnancy and calling for urgent attention and action that is helpful to prevent the resulting adverse birth outcomes together with stillbirth (30).

However, there is no specific preventive strategic plan to reduce and control the existence of stillbirth; Ethiopia set different maternal and child healthcare plans like SDP (31) to combat such problems (32). Studies to identify the magnitude and determinants of stillbirth among diabetic pregnant mothers in Ethiopia had great discrepancy and inconsistent finding. Therefore, this systematic review and meta-analysis was the first in its kind to assess the pooled prevalence of stillbirth and its determinants among diabetic pregnant mothers in Ethiopia, which is pivotal to plan, evaluate, and improve national preventive measures of stillbirth among diabetic pregnant mothers.

## Methods

### Reporting

The review protocol has been registered in the international prospective register of systematic reviews (PROSPERO) with registration number (PROSPERO 2020: CRD2020167734), and the result of the review was presented based on standard Preferred Reporting Items for Systematic review and Meta-analysis (PRISMA) checklist (33) (Supporting Information 1).

### Inclusion and Exclusion Criteria

Cross-sectional, case–control, and prospective cohort studies that reported the magnitude of stillbirth and/or at least one determinant among diabetic pregnant mothers were included. Similarly, both published and unpublished studies were involved. Studies that were conducted among pregnant women with pre-existing (type 1 and 2 diabetes mellitus) and/or gestational diabetes mellitus confirmed by WHO were included. However, articles without full-text or abstract were excluded.

### Outcomes of Interest

Stillbirth among diabetic pregnant mothers is the primary outcomes of interest; it can be defined as the birth of an infant at or after 28 weeks of gestation who at the time of delivery did not breathe or show signs of life (8).

### Electronic Search

Electronic databases such as PubMed, Web of Science, Scopus, Google Scholar, and ScienceDirect were searched using Population, Intervention, Comparison and Outcomes (PICO) format. The included search terms used a combination of relevant Medical Subject Heading (MeSH) Boolean operators, such as “AND” and “OR,” to combine search terms. The search strategy was started on October 16, 2020, and an updated search was conducted on November 2, 2020, using the following search terms: (pregnancy OR pregnant) AND (women OR mothers) AND (diabetes mellitus OR type 1 diabetes mellitus OR juvenile diabetes mellitus OR insulin-dependent diabetes mellitus OR gestational diabetes mellitus OR type 2 diabetes mellitus OR adult-onset diabetes mellitus OR non-insulin dependent diabetes mellitus OR pre-existing diabetes mellitus OR pre-gestational diabetes mellitus) AND (fetal loss OR still birth) AND (Ethiopia). Moreover, gray literature found in Addis Ababa and Haramaya University online repositories was used. In addition, reference lists and citations of included papers were cheeked to identify other potentially relevant articles. Finally, the result of search strategy for PubMed database had been presented as an example (Table 1).

**Table 1:** Search strategy for PubMed data base.

**Research question: Do pregnant women who have diabetes compared to non-diabetes pregnant women have higher stillbirth?**				
**Data base**	**Search date**	**Search**	**Query**	**Results**
PubMed	Nov 2/2020	#1& #2 & #3 & #4	Search: (((((((((((((((((pregnancy[MeSH Terms]) OR (pregnant[MeSH Terms])) AND (mothers[MeSH Terms])) OR (women[MeSH Terms])) AND (diabetes mellitus[MeSH Terms])) OR (type 1 diabetes mellitus[MeSH Terms])) OR (type 2 diabetes mellitus[MeSH Terms])) OR (juvenile diabetes mellitus[MeSH Terms])) OR (insulin-dependent diabetes mellitus[MeSH Terms])) OR (gestational diabetes mellitus[MeSH Terms])) OR (adult-onset diabetes mellitus[MeSH Terms])) OR (non- insulin dependent diabetes mellitus[MeSH Terms])) OR (pre-existing diabetes mellitus[MeSH Terms])) OR (pre-gestational diabetes mellitus[MeSH Terms])) AND (fetal loss[MeSH Terms])) OR (stillbirths[MeSH Terms]))) AND (Ethiopia)	57
		#4	Search: (Fetal loss [MeSH Terms]) OR (stillbirth [MeSH Terms])	4,984
		#3	Search: (((((((((diabetes mellitus[MeSH Terms]) OR (type 1 diabetes mellitus[MeSH Terms])) OR (juvenile diabetes mellitus[MeSH Terms])) OR (insulin-dependent diabetes mellitus[MeSH Terms])) OR (gestational diabetes mellitus[MeSH Terms])) OR (type 2 diabetes mellitus[MeSH Terms])) OR (adult-onset diabetes mellitus[MeSH Terms])) OR (non- insulin dependent diabetes mellitus[MeSH Terms])) OR (pre-existing diabetes mellitus[MeSH Terms])) OR (pre-gestational diabetes mellitus [MeSH Terms])	431,809
		#2	Search: (Women) OR (mothers)	1,569,569
		#1	Search: (Pregnancy [MeSH Terms]) OR (pregnant [MeSH Terms])	902,064

Population, Intervention, Comparison, and Outcomes (PICO) were as follows: P: pregnant women, I: diabetes, C: non-diabetes, O: stillbirth.

Research question: Do pregnant women who have diabetes compared to non-diabetic pregnant women have higher stillbirth?

### Study Selection and Quality Assessment

EndNote version 9 (Thomson Reuters, London) reference manager (34) was used to remove duplicate studies. After screening titles and abstracts, a full-text review was done to determine the eligibility of each study by two independent authors (DM and WA). Any discrepancy was resolved by a third author (WA). Elsewhere, two independent authors (DM and DK) evaluated the eligibility of all retrieved studies using the Joanna Briggs Institute (JBI) quality appraisal checklist (35). Hence, one prospective cohort study (18), four cross-sectional studies (19–21), and two case–control studies (23, 36) were appraised for quality using the JBI checklist. Therefore, studies are considered high quality whenever they meet 50% and/or above quality assessment criteria. Accordingly, none of these studies were excluded based on the quality assessment criteria as the quality assessment result ranges from 62.5 to 100% (Table 2).

**Table 2:** Quality of included studies using Joanna Briggs Institute critical Appraisal Tool for cross-sectional, case-control, and prospective cohort.

**Quality of included studies using Joanna Briggs Institute critical Appraisal Tool for cross-sectional**					
**No**	**Criteria**	**Bajrond et al**. **(****21****)**	**Selamawit et al**. **(****22****)**	**Abdisa et al**. **(****19****)**	**Zewedu G (** **20** **)**
1	Were the criteria for inclusion in the sample clearly defined?	Yes	Yes	Yes‘	No
2	Were the study subjects and the setting described in detail?	Yes	Yes	Yes	Yes
3	Was the exposure measured in a valid and reliable way?	Yes	Yes	Yes	No
4	Were objective, standard criteria used for measurement of the condition?	Yes	Yes	Yes	No
5	We're confounding factors identified?	Yes	Yes	Yes	Yes
6	Were strategies to deal with confounding factors stated?	No	Yes	No	Yes
7	Were the outcomes measured in a valid and reliable way?	Yes	Yes	Yes	Yes
8	Was appropriate statistical analysis used?	Yes	Yes	Yes	Yes
Percentage of yes (%)		7/8 = 87.5%	8/8 = 100%	8/8 = 100%	5/8 = 62.5%
**Quality of included studies using Joanna Briggs Institute Critical Appraisal Tool for case-control**					
**No**	**Criteria**	**Elias et al**. **(****23****)**	**Abay et al**. **(****36****)**		
1	Were the groups comparable other than the presence of disease in cases or the absence of disease in controls?			Yes	Yes
2	Were cases and controls matched appropriately?			No	No
3	Were the same criteria used for identification of cases and controls?			No	No
4	Was exposure measured in a standard, valid and reliable way?			Yes	Yes
5	Was exposure measured in the same way for cases and controls?			Yes	Yes
6	We're confounding factors identified?			Yes	Yes
7	Were strategies to deal with confounding factors stated?			Yes	Yes
8	Were outcomes assessed in a standard, valid and reliable way for cases and controls?			Yes	Yes
9	Was the exposure period of interest long enough to be meaningful?			No	No
10	Was appropriate statistical analysis used?			Yes	Yes
Percentage of yes (%)				7/10 = 70%	7/10 = 70%
**Quality of included studies using Joanna Briggs Institute Critical Appraisal Tool for prospective cohort**					
No	**Criteria**			**Talema et al**. **(****18****)**	
1	Were the two groups similar and recruited from the same population?			No	
2	Were the exposures measured similarly to assign people to both exposed and unexposed groups?			Yes	
3	Was the exposure measured in a valid and reliable way?			Yes	
4	We're confounding factors identified?			Yes	
5	Were strategies to deal with confounding factors stated?			Yes	
6	Were the groups/participants free of the outcome at the start of the study (or at the moment of exposure)?			Yes	
7	Were the outcomes measured in a valid and reliable way?			Yes	
8	Was the follow up time reported and sufficient to be long enough for outcomes to occur?			Yes	
9	Was follow up complete, and if not, were the reasons to loss to follow up described and explored?			No	
10	Were strategies to address incomplete follow up utilized?			Yes	
Percentage of yes (%)				8/10 = 80%	

### Risk of Bias Assessment

The risk of bias tool developed by Hoy et al. (37) was used to assess the risk of bias for each study. The tool comprises 10 items that address four areas of bias. Items 1–4 assess the external validity of the studies (domains are selection and non-response bias) and items 5–10 assess the internal validity of the studies (items 5–9 assess the domain of measurement bias, and item 10 assesses bias related to analysis). Accordingly, studies were classified as having a low risk of bias when 8 or more of the 10 questions were answered “yes,” a moderate risk of bias when 6 to 7 of the 10 questions were answered “yes,” and a high risk of bias when 5 or fewer questions were answered “yes” (Table 3).

**Table 3:** Result of risk of bias assessment.

**No**.	**Item**	**Talema et al. (18)**	**Elias et al. (23)**	**Bajrond et al. (21)**	**Selamawit et al. (22)**	**Abdisa et al. (19)**	**Abay et al. (36)**	**Zewedu G (20)**.
**EXTERNAL VALIDITY**								
1	Was the study's target population a close representation of the national population in relation to relevant variables?	No	No	No	No	No	No	No
2	Was the sampling frame a true or close representation of the target population?	Yes	Yes	Yes	Yes	Yes	Yes	Yes
3	Was some form of random selection used to select the sample, OR was a census undertaken?	Yes	Yes	Yes	Yes	Yes	Yes	Yes
4	Was the likelihood of nonresponse bias minimal?	Yes	Yes	Yes	Yes	Yes	Yes	Yes
**INTERNAL VALIDITY**								
5	Were data collected directly from the subjects (as opposed to a proxy)?	Yes	Yes	Yes	Yes	Yes	Yes	Yes
6	Was an acceptable case definition used in the study?	Yes	Yes	Yes	Yes	Yes	Yes	Yes
7	Was the study instrument that measured the parameter of interest shown to have validity and reliability?	Yes	Yes	Yes	Yes	Yes	Yes	Yes
8	Was the same mode of data collection used for all subjects?	Yes	Yes	Yes	Yes	Yes	Yes	Yes
9	Was the length of the shortest prevalence period for the parameter of interest appropriate?	No	No	No	Yes	No	Yes	Yes
10	Were the numerator(s) and denominator(s) for the parameter of interest appropriate?	Yes	Yes	Yes	Yes	Yes	No	Yes
	No of yes	8	8	8	9	8	8	8
11	Summary item on the overall risk of study bias	Low risk	Low risk	Low risk	Low risk	Low risk	Low risk	Low risk

### Data Extraction

After collecting the required articles from the entire database, all important data were extracted by the authors (AY and AS) using the standardized data extraction form and cross-checked to ensure consistency. Three authors (DM, WA, and DK) extracted data independently on author/s name, year of publication, study area/region, study design, sample size, and prevalence of stillbirth with 95% CI and determinants. Any dissimilarity and inconsistencies among the authors were resolved by discussion and repeating the procedure. The reviewer contacted the corresponding author for further information whenever pertinent data were missed from the included studies.

### Data Analysis

The extracted data were exported to Stata version 14 statistical software for meta-analysis. *I*^2^ statistic and Cochran's “*Q*” test was used to calculate the percentage of total variation in the study estimated due to heterogeneity. *I*^2^ statistic ranged between 0 and 100%; the value of 0, 25, 50, and 75% represented no, low, moderate, and high heterogeneity, respectively. In addition, a *p*-value of the *I*^2^ statistic of <0.10 was used to declare significant heterogeneity (38, 39). The pooled prevalence of stillbirth with 95% CI was estimated using a fixed-effect model in the absence of any significant heterogeneity. On the other hand, the random-effect model was used if the total variation across studies was significant. A forest plot was used to show the effect sizes of selected independent factors using AOR with 95% CI. A funnel plot was used to cheek the presence of publication bias (40). Egger's test was used to determine the presence of significant publication bias. An Egger's test *p*-value of <0.05 was considered to declare significant publication bias (41). Lastly, trim and fill analysis was performed after Egger's test, suggesting the presence of publication bias.

## Results

### Search Results and Study Characteristics

**Figure 1:**
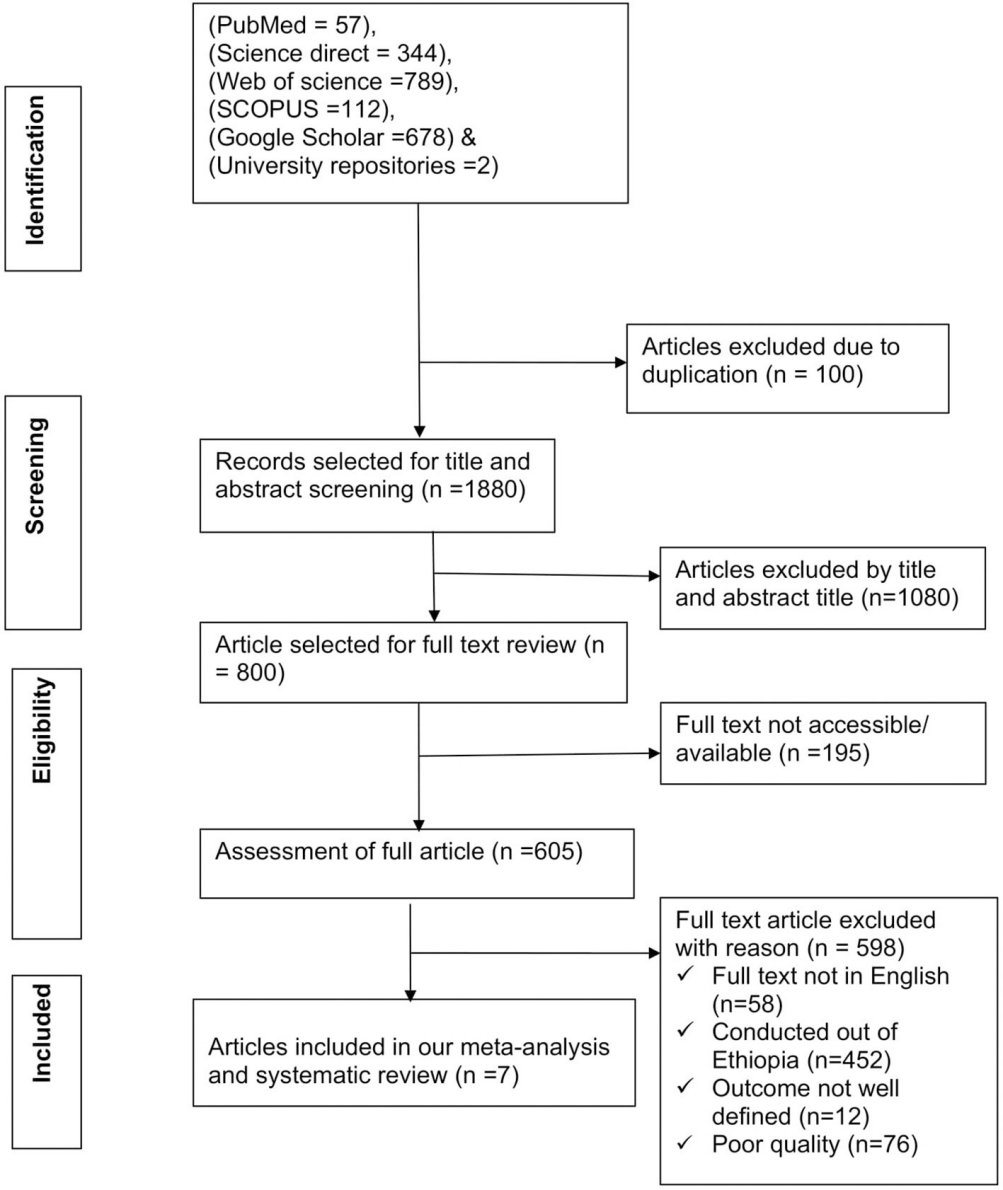
PRISMA flow diagram.

Our searches from different electronic databases yielded a total of 1,982 articles. After removing 100 duplicate articles, 1,880 articles remained. From the remaining 1,880 articles, 1,080 articles were excluded after reviewing titles and abstracts. From the remaining 800 articles, 195 articles were omitted because their full text was not available. Lastly, 605 articles were suitable for full-text review. Still, 598 articles were excluded based on the predetermined eligibility criteria. Finally, seven articles (18–23, 36) were included for meta-analysis (Figure 1).

The total sample size of the included studies was 1,225 diabetes mellitus in pregnancy, where the sample size of the individual studies ranges from 45 in Hiwot Fana and Dilchora Hospital (23) to 346 in Mettu Karl Hospital (19). From the included studies, four were done in Addis Ababa (18, 20–22), one study was carried out in Amhara region (36), and the remaining two were conducted in the Oromoia region (19, 42). All studies were conducted at different hospitals of Ethiopia using different study designs: four cross-sectional (19–21), two case-control (23, 36), and one prospective cohort (18). Moreover, all the included studies were studied from the period 2013–2018. Among the included studies, six studies contributed data for the prevalence of stillbirth (18–23) and five studies (18, 19, 21, 22, 36) contributed data regarding determinants of stillbirth with response rate ranges from 80.2 to 100%. Accordingly, the prevalence of stillbirth among diabetic pregnant women ranged from 2.6 to 16.05% (19, 22). Moreover, the included studies showed that the presence of glucometer at home, preconception care, being a housewife, high blood glucose level, antenatal care (ANC) visit, maternal age, rural residency, short birth interval, level of education, and history of previous adverse birth outcomes were significantly associated with stillbirth (18, 19, 21, 22, 36) (Table 4).

**Table 4:** Study characteristics included in systematic review and meta-analysis of stillbirth and determinants among diabetes mothers in Ethiopia, 2020.

**Authors**	**Area**	**Study design**	**Sample size**	**Prevalence**	**Response rate (%)**	**Outcome variable**	**Factors**	**Quality**
Talema A et al. (1)	Teaching hospital in Addis Ababa	Prospective cohort	80	3.75	100	Stillbirth	Pre-conception care and home glucometer	Low risk
Elias et al. (2)	Hiwot Fana and Dilchora Hospital, Oromoia	Un matched case-control	45	8.89	100	Stillbirth	Not reported	Low risk
Bajrond E et al. (3)	Tikur Anbessa Hospital, Addis Ababa	Retrospective cross-sectional	337	2.67	100	Stillbirth	House wife and preterm delivery	Low risk
Selamawit E et al. (4)	Tikur Anbessa Hospital, Addis Ababa	Retrospective cross-sectional	162	16.05	80.20	Stillbirth	Maternal age and blood glucose level	Low risk
Abdisa B et al. (5)	Mettu Karl Hospital, Oromoia	Retrospective cross-sectional	346	2.60	95.60	Stillbirth	House wife and preterm delivery	Low risk
Abay W et al. (6)	Dessie, Debre Birhan and Bahir Dar hospital, Amhara	Un matched case-control	134	0.00	97.10	–	Rural, Illiteracy, no ANC, adverse birth outcomes, short birth spacing, and maternal age	Low risk
Zewedu G (7)	Selected hospital in Addis Ababa	Cross-sectional	111	2.70	100	Stillbirth	Not reported	Low risk

### Meta-Analysis

**Figure 2:**
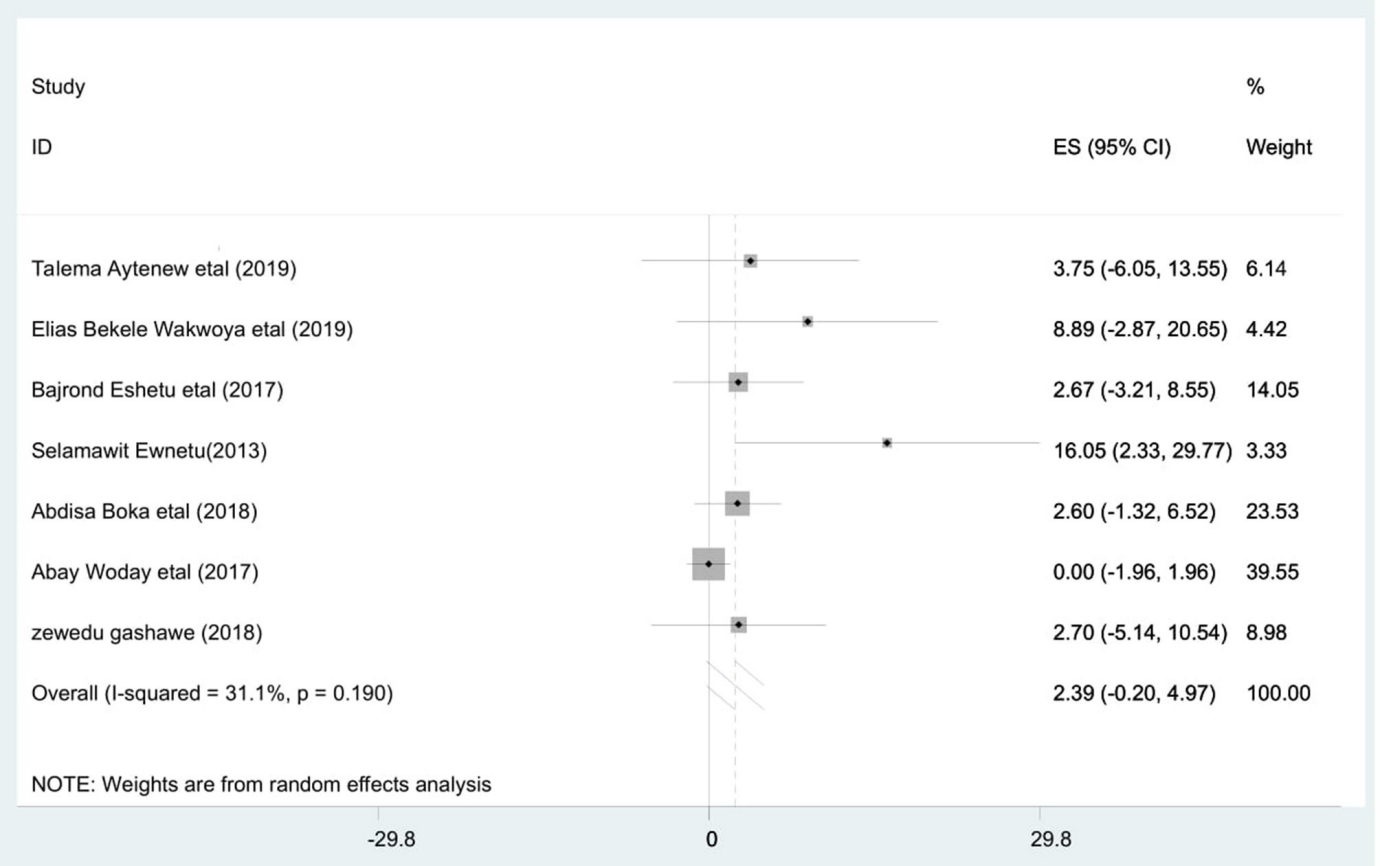
Forest plot of pooled prevalence of stillbirths among diabetic mothers in Ethiopia, 2020.

The highest prevalence of stillbirth among diabetic pregnant mothers was reported in Tikur Anbessa Hospital, Addis Ababa (16.05%) (22) whereas the least was observed in Mettu Karl Hospital, Oromoia region (2.6%) (19). However, the pooled prevalence of stillbirth among diabetic pregnant mothers was 2.39 [95% CI: −0.20, 4.97; *I*^2^ = 31.1%, *p* = 0.19]. This shows that the included studies had moderate heterogeneity (*I*^2^ = 31.1%, *p* = 0.19) (Figure 2).

### Publication Bias

**Figure 3:**
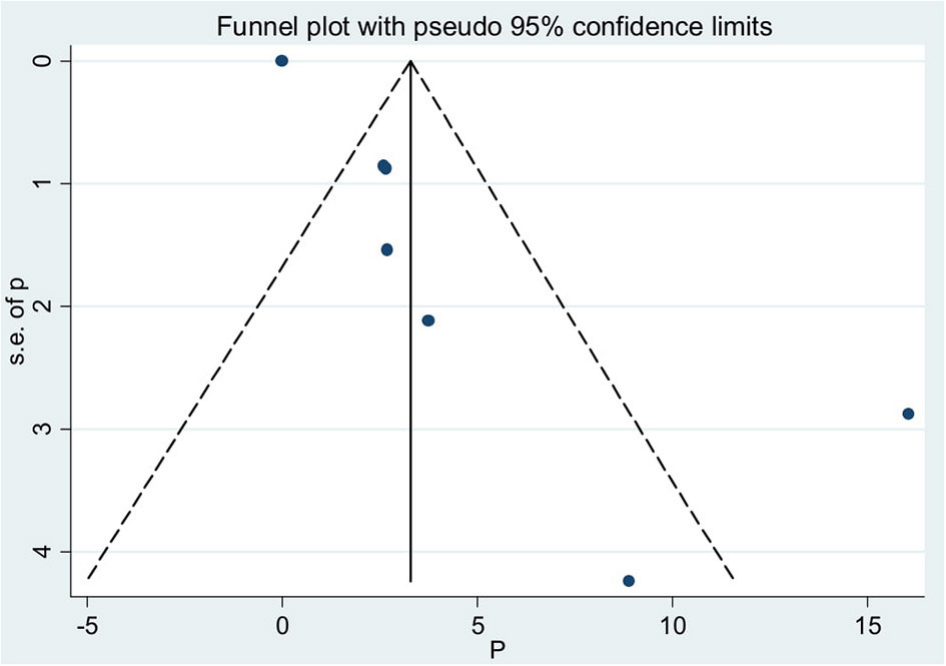
Funnel plot to show publication bias for stillbirths among diabetic mothers in Ethiopia, 2020.

Asymmetrical distribution of the funnel plot implies the presence of publication bias among the included studies (Figure 3). Furthermore, Egger's test with a *p*-value of 0.006 shows the presence of publication bias (Table 5).

**Table 5:** Egger's test to show publication bias.

**Outcomes**	**Std_Eff**	**Coef**.	**Std. Err**.	***t***	***P*** **> |*t*|**	**95% CI**
Stillbirths	Slope	–1.63	0.78	–2.08	0.09	–3.64 0.39
	Bias	1.66	0.36	4.56	0.00	0.72 2.59

### Trim and Fill Analysis

We have performed trim and fill method analysis. A bias-adjusted effect estimate of stillbirth among diabetic pregnant mothers was found to be 1.104 at a *p*-value of 0.420, assuming there are missing studies (Table 6).

**Table 6:** Trim and fill analysis of stillbirth among diabetic women in Ethiopia, 2020 Meta-analysis.

**Method**	**Pooled Est**.	**95% CI**		**Asymptotic**		**No. of studies**
		**Lower**	**Upper**	***z*** **-Value**	***p*** **-Value**	
Fixed	1.22	–0.377	2.81	1.50	0.14	7
Random	2.39	–0.201	4.97	1.81	1.81	
Test for heterogeneity: Q = 8.714 on 6 degrees of freedom (*p* = 0.190).						
Moment-based estimate of between studies variance = 3.407.						
Trimming estimator: Linear.						
Meta-analysis type: Fixed-effects model.						
**Iteration**	**Estimate**	**Tn**	**# to trim**		**Diff**	
1	1.22	27	4		28	
2	0.70	27	4		0	
Filled						
Meta-analysis						
**Method**	**Pooled Est**.	**95% CI**		**Asymptotic**		**No. of studies**
		**Lower**	**Upper**	***z*** **-value**	***p*** **-value**	
Fixed	0.70	−0.83	2.22	0.90	0.37	11
Random	1.10	−1.58	3.79	3.79	0.42	

*Test for heterogeneity: Q = 16.426 on 10 degrees of freedom (p = 0.088). Moment-based estimate of between studies variance = 6.375*.

### Investigation of Heterogeneity

There was no significant heterogeneity among studies to show the pooled prevalence of stillbirth (*I*^2^ = 31%, *p* = 0.19).

### Determinants of Stillbirth

**Figure 4:**
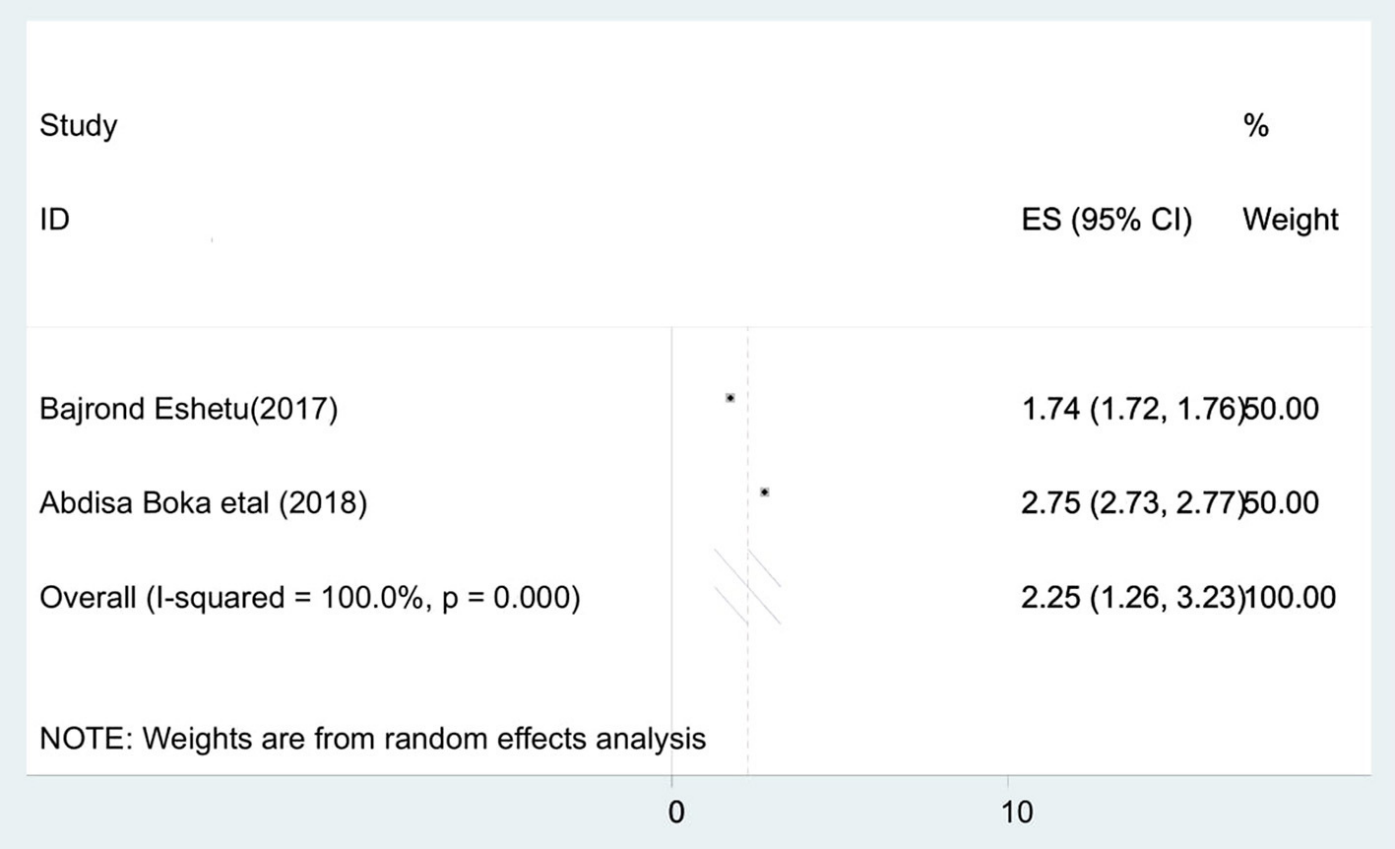
Forest plot to show the association between housewife and pooled prevalence of stillbirths among diabetic pregnant mothers in Ethiopia, 2020.

**Figure 5:**
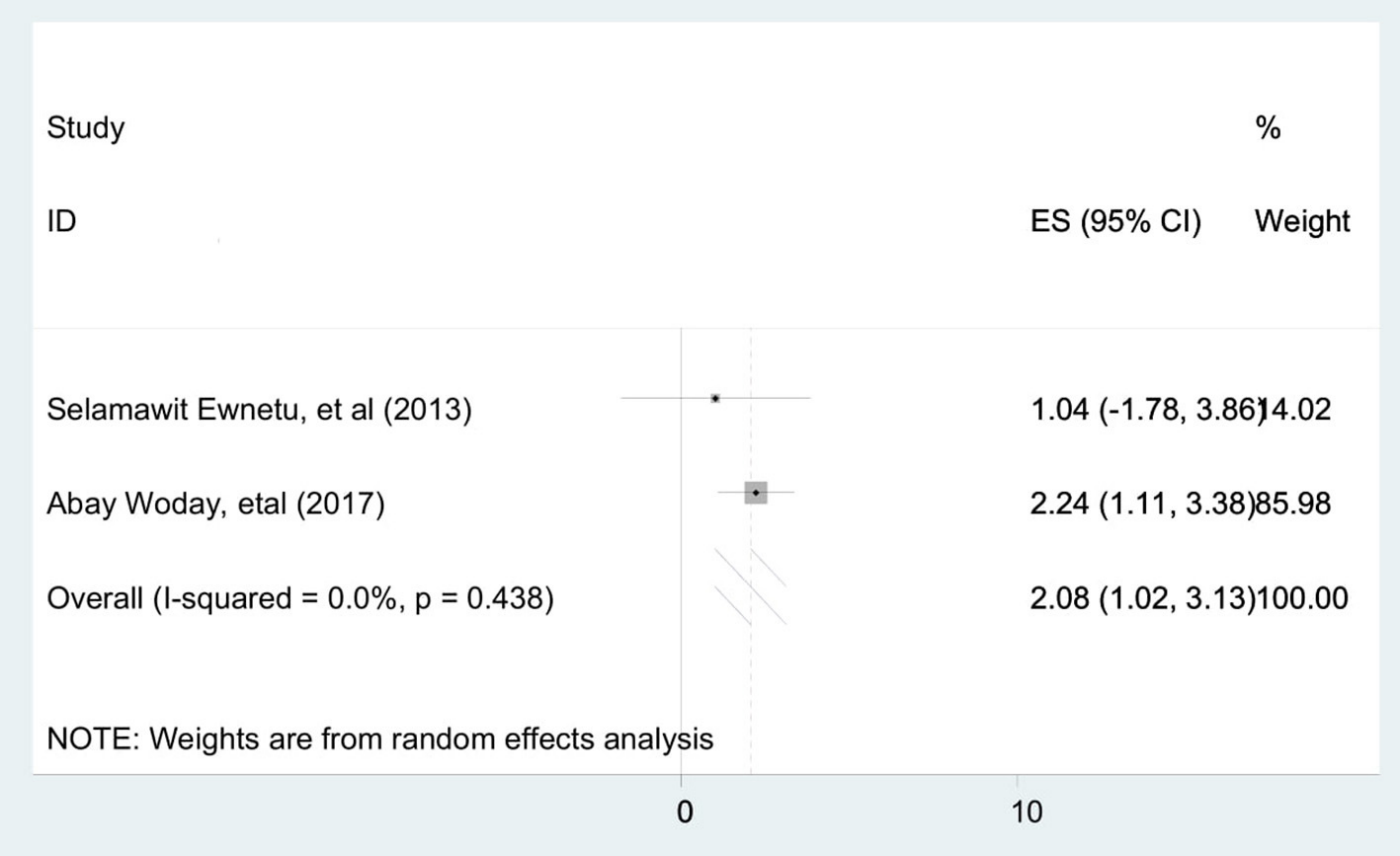
Forest plot to show the association between maternal age of <30 years and pooled prevalence of stillbirths among diabetic pregnant mothers in Ethiopia, 2020.

**Figure 6:**
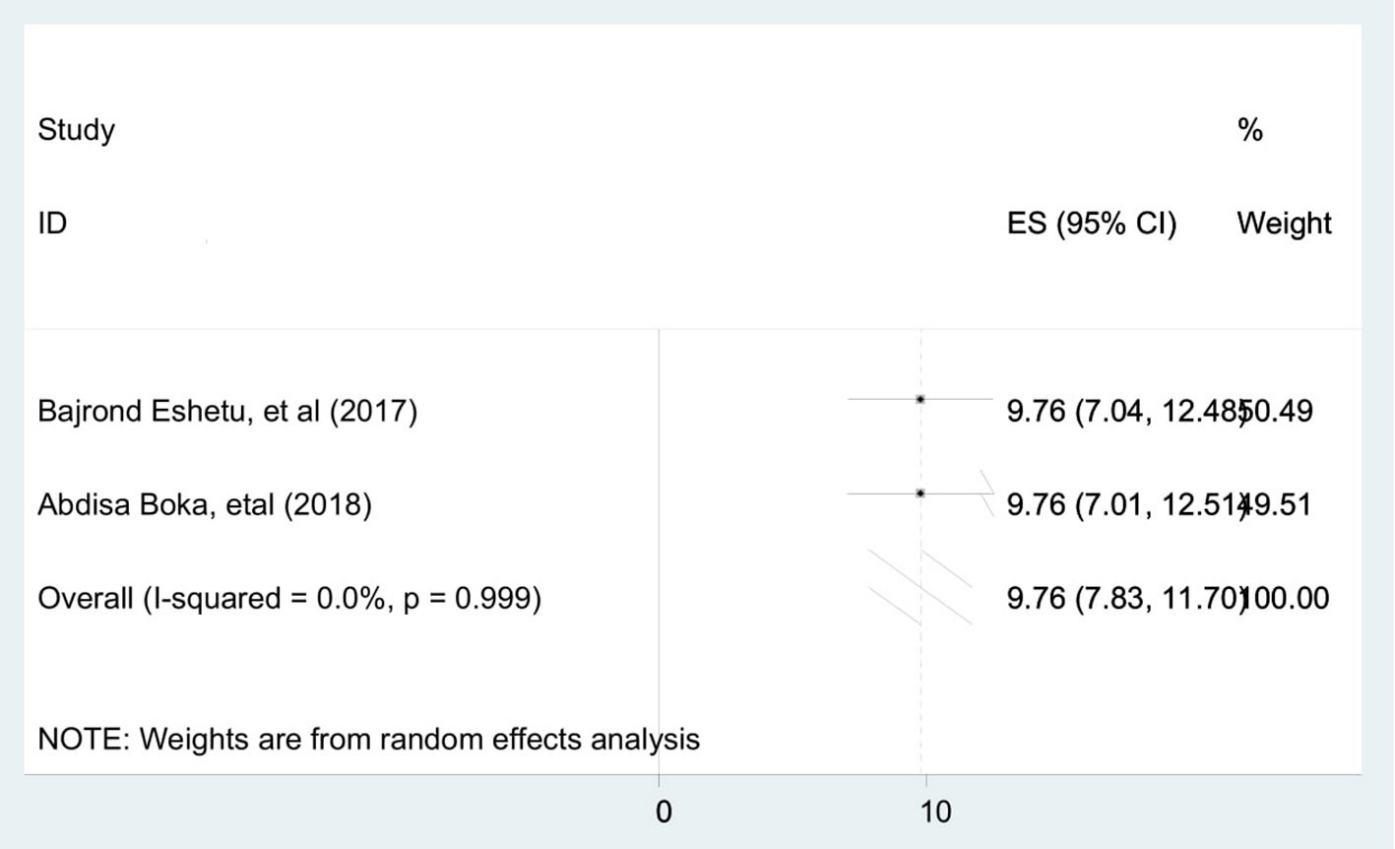
Forest plot to show the association between gestational age of <37 completed weeks and pooled prevalence of stillbirths among diabetic pregnant mothers in Ethiopia, 2020.

To identify the pooled determinants for the prevalence of stillbirth among diabetic pregnant mothers, meta-analysis was computed. We only included adjusted factors that were investigated in at least two studies, and the definition of the same factors has to be similar across all the included studies. As a result, a maternal age of <30 years, a gestational age of <37 completed weeks, and being a housewife were the criteria involved in the meta-analysis. Consequently, being a housewife [AOR = 2.25 (95% CI: 1.26, 3.23)] (Figure 4), maternal age of <30 years [AOR = 2.08 (95% CI: 1.02, 3.13)] (Figure 5), and gestational age of <37 completed weeks [AOR = 9.76 (95% CI: 7.83, 11.70)] (Figure 6) were significantly associated with stillbirth among diabetic pregnant mothers.

## Discussion

Increased risk of stillbirth in diabetes pregnancies has been a well-known and recognized complication for decades. This systematic review and meta-analysis estimated the pooled prevalence and determinants of stillbirth among diabetic pregnant mothers in Ethiopia. Hence, the pooled prevalence of stillbirth among diabetic pregnant mothers was 2.39 [95% CI: −0.20, 4.97], which is consistent with the study conducted in King Khalid University Hospital (2.6%) (17), Saudi Arabia (3.66%) (14), Delhi (2.6%) (43), and Vietnam (1.43%) (44). This consistency could be due to a similar healthcare package and system toward maternal and newborn health service between countries.

However, our finding is lower than the finding from Ghana 5.55% (16), Canada (13.3%) (45), and China (42.7%) (46). Differences in universal screening strategies of diabetes mellitus during pregnancy between the countries contributed for the discrepancy; Ethiopia has been using WHO diabetes screening strategies, whereas Ghana and China has been using American Diabetes Association criteria to screen diabetes mellitus during pregnancy. Besides, the difference in the study participants between Canada (only women with pre-existing diabetes mellitus) and Ethiopia (both pre-existing and gestational diabetes mellitus) might have played a role for the variation.

However, our finding is higher than the study conducted in Bangladesh (0.014%) (47) and England (13.9 per 1,000 births) (12). The possible reason for the variation could be due to the socio-demographic difference of the study participants. The study participants in Ethiopia were from both rural and urban residency. In contrast, the study participants in Bangladesh were only from urban areas. Furthermore, the outcome of interest in Ethiopia was after 28 weeks of gestation whereas that in England was after 32 weeks of gestation.

Based on this review, the risk of stillbirth among diabetic pregnant housewife mothers was 2.25 times higher than their counterpart. This is due to the fact that housewife mothers have a low socio-economic status and have limited information about the importance of ANC to reduce the risk of adverse birth outcomes like stillbirth.

Likewise, the likelihood of stillbirth among diabetic mothers <30 years of age was two times higher than their counterpart. This is due to null parity being a risk factor for stillbirth. Thus, a low maternal age mother probably gives stillbirth due to null parity.

Moreover, the odds of stillbirth among diabetic mothers with a gestational age of <37 completed weeks was 9.76 times higher than the respective counterpart. This is due to the fact that when gestational age decreases, the maturity of the fetus and the chance to survive decrease, which results in stillbirth.

It is recommended to offer concern for mothers with low maternal age during pregnancy. It is also highly recommended to increase the coverage of ANC for all mothers with hyperglycemia for early detection and to control glycemia to normal levels. Furthermore, community awareness about the related adverse birth outcomes due to maternal hyperglycemia should be promoted.

## Strength and Limitations of the Study

This is the first systematic review and meta-analysis to estimate the national prevalence of stillbirth and its determinants among diabetic mothers. The limited number of primary studies used to investigate the pooled prevalence of stillbirth in the nation is a limitation. Besides, all the primary studies are hospital-based studies, which affects representativeness to the general community.

## Conclusions

The national pooled prevalence of stillbirth among diabetic pregnant mothers was 2.39%, which is higher than the prevalence of stillbirth among the general population (1.18%). Low maternal age, gestational age of <37 completed weeks, and being a housewife were significant determinants of stillbirth.

## Data Availability Statement

The original contributions presented in the study are included in the article/Supplementary Material, further inquiries can be directed to the corresponding author/s.

## Author Contributions

DB, WB, AYA, ASA, DM, and BB developed the protocol and were involved in the design, selection of study, data extraction, quality assessment, statistical analysis, developing the initial drafts of the manuscript, and revising the subsequent draft. All authors prepared, read, and approved the final draft of the manuscript.

## Conflict of Interest

The authors declare that the research was conducted in the absence of any commercial or financial relationships that could be construed as a potential conflict of interest.

## Publisher's Note

All claims expressed in this article are solely those of the authors and do not necessarily represent those of their affiliated organizations, or those of the publisher, the editors and the reviewers. Any product that may be evaluated in this article, or claim that may be made by its manufacturer, is not guaranteed or endorsed by the publisher.

## Supplementary Material

Additional file 1 PRISMA checklist.

## Abbreviations

ANC: antenatal care
AOR: adjusted odds ratio
CI: confidence interval
FIGO: International Federation of Obstetrics and Gynecology
IDF: International Diabetic Federation
JBI: Joanna Briggs Institute
LMICs: low- and middle-income countries
PRISMA: Preferred Reporting Items for Systematic Review and Meta-analysis
SDP: Sustainable Development Plan
WHO: World Health Organization.
